# Evidence from molecular dynamics simulations of conformational preorganization in the ribonuclease H active site

**DOI:** 10.12688/f1000research.3605.1

**Published:** 2014-03-07

**Authors:** Kate A. Stafford, Arthur G. Palmer III

**Affiliations:** 1 Department of Biochemistry and Molecular Biophysics, Columbia University, New York, NY 10032, USA

## Abstract

Ribonuclease H1 (RNase H) enzymes are well-conserved endonucleases that are present in all domains of life and are particularly important in the life cycle of retroviruses as domains within reverse transcriptase. Despite extensive study, especially of the E. coli homolog, the interaction of the highly negatively charged active site with catalytically required magnesium ions remains poorly understood. In this work, we describe molecular dynamics simulations of the E. coli homolog in complex with magnesium ions, as well as simulations of other homologs in their apo states. Collectively, these results suggest that the active site is highly rigid in the apo state of all homologs studied and is conformationally preorganized to favor the binding of a magnesium ion. Notably, representatives of bacterial, eukaryotic, and retroviral RNases H all exhibit similar active-site rigidity, suggesting that this dynamic feature is only subtly modulated by amino acid sequence and is primarily imposed by the distinctive RNase H protein fold.

## Introduction

**Figure 1.**
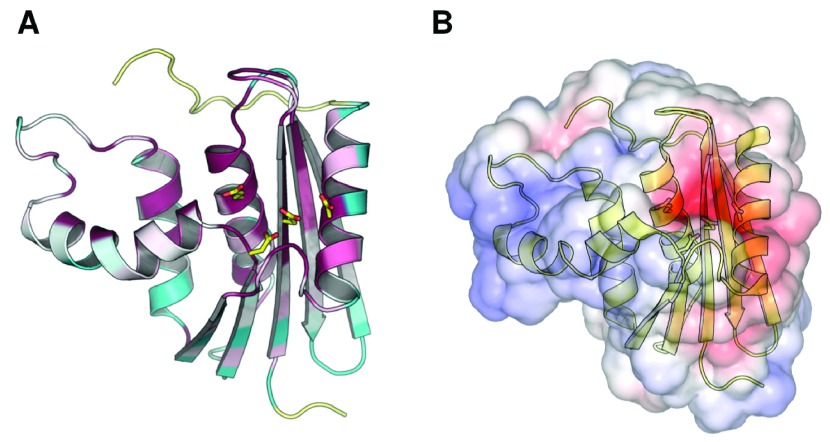
Conservation of the ribonuclease H active site. ( **A**) Residue conservation among bacterial RNase H homologs mapped onto the structure of ecRNH (PDB ID 2RN2). Highly conserved residues are shown in red, highly variable residues in green, and sites with insufficient data in yellow. Image produced using ConSurf. ( **B**) Electrostatic map of the solvent-accessible surface of ecRNH produced using APBS. Red represents regions of negative charge and blue represents regions of positive charge. The active-site residues are represented as sticks in both cases.

Ribonuclease H1 (RNase H) proteins are well-conserved endonucleases that are found in all domains of life and cleave the RNA strand of an RNA-DNA duplex substrate. The RNase H active site canonically consists of a highly conserved DED(D) motif ( Figure 1), three to four carboxylate-containing residues collectively participating in the binding of catalytically required divalent cations, Mg ^2+^ under physiological conditions. This active-site sequence motif and requirement for Mg ^2+^ is widely shared with other nucleases, suggesting a common catalytic mechanism ^1^.

**Figure 2.**
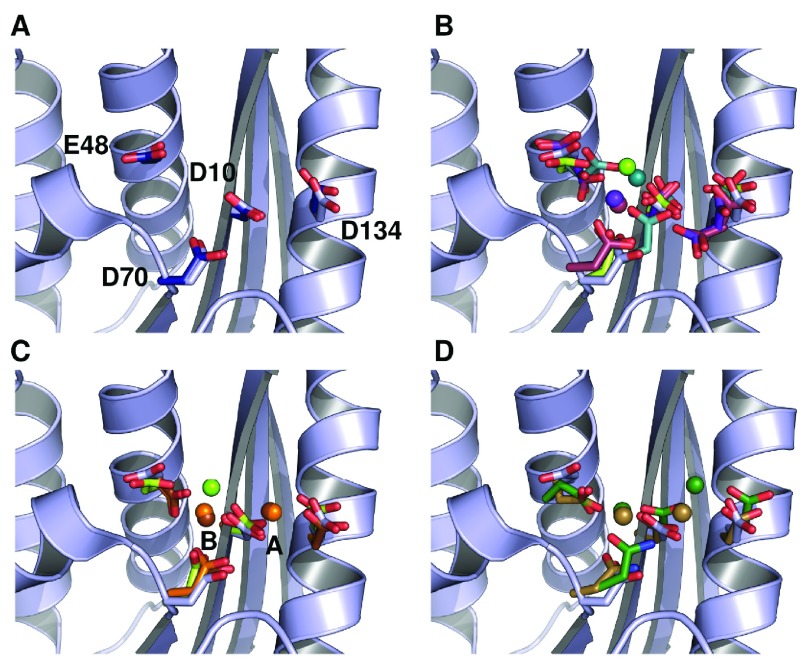
Conformational diversity of metal-ion interactions with ecRNH as determined by crystallography. In all cases the backbone and active-site sidechains from ecRNH in the absence of ion (PDB ID 2RN2) are shown in light blue for comparison. ( **A**) Structural superposition of the four active-site residues in two structures of ecRNH in the absence of metal ions: 2RN2 (light blue) and 1RNH (dark blue). ( **B**) Structural diversity of RNases H in complex with a single Mg ^2+^ ion: ecRNH (1RDD), green; XMRV WT (4E89), dark cyan; XMRV ∆C (3P1G), maroon; MoMLV ∆C (2HB5), purple. The two deletion mutants (indicated as ∆C) bind Mg ^2+^ in slightly different positions than do their corresponding full-length proteins and both contain two alternate conformations for E48 and D134. ( **C**) Comparison of Mg ^2+^ and Mn ^2+^ complexes: ecRNH with Mg ^2+^ (1RDD), green; ecRNH with Mn ^2+^ (1G15), orange; HIV RNase H domain with ecRNH helix C insertion with Mn ^2+^ (3HYF), brown. ( **D**) Structural diversity of RNases H in complex with substrate: *Homo sapiens* RNase H with Ca ^2+^ ions (2QKK), brown; *Bacillus halodurans* RNase H with Mn ^2+^ ions (1ZBI), dark green.

The best-studied member of the RNase H family is the homolog from *Escherichia coli* (ecRNH), in which this active-site motif is represented as D10, E48, D70, and D134 ^2^ ( Figure 2A). Measurements of the pKa values of the active-site residues indicate perturbed pKa values for D10 and D70 which normalize upon Mg ^2+^ binding, clearly establishing these residues as critical for interaction with ions ^3^. The pH optimum for the RNase H reaction *in vitro* is approximately 7.5–8.5 ^4^, a value at which all active-site residues should be deprotonated ^3^ and therefore accessible for ion binding.

Despite extensive study, the interaction of metal ions with the ecRNH active site is poorly understood. Activity has been reported in the presence of Mn ^2+^ as well as the physiologically relevant Mg ^2+^ ^5^. Significant differences have been observed between the protein’s interactions with Mg ^2+^ and Mn ^2+^. Co-crystallization studies of ecRNH with high concentrations of Mg ^2+^ find a single bound metal ion ^6^ ( Figure 2B). By contrast, co-crystallization with Mn ^2+^ reveals two bound ions, one associated with residues D10 and D134 (denoted the A site), and one associated with D10, E48, and D70 (denoted the B site) ^7^ ( Figure 2C); the B site is similar but not identical to the previously identified Mg ^2+^ site. Single Mn ^2+^ sites have been identified in the presence of mutations of E48 and/or D134 ^8^, both of which are dispensable for Mn ^2+^-dependent activity ^9^. Crystallographic studies of related RNases H from the archaeal extremophile *Bacillus halodurans* ^10^ and from *Homo sapiens* ^11^ in complex with substrate find two bound ions in the active site ( Figure 2D).

**Figure 3.**
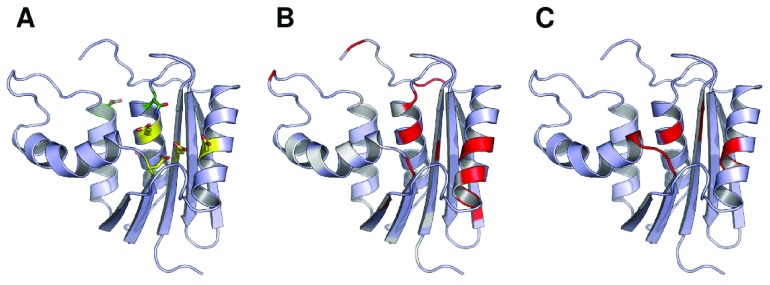
Experimental NMR measurements of the effects of Mg ^2+^ binding mapped onto the structure of ecRNH. ( **A**) Active site residues (yellow) and other DENQ residues (green) that experience perturbation upon Mg ^2+^ binding ^24^. ( **B**) Chemical shift perturbation values for sidechain *C ^γδ^* reflecting the effects of Mg ^2+^ binding ^24^. White corresponds to no chemical shift change, red corresponds to a large change, and non-DENQ residues are shown in light blue. ( **C**) Residues previously shown to experience backbone ^15^N or ^1^H chemical shift changes upon binding Mg ^2+^ ^25^.

Experimental evidence from nuclear magnetic resonance (NMR) studies locates the area surrounding the active site as the region most susceptible to perturbation upon interaction with ions ( Figure 3). Titration of Mg ^2+^ with ecRNH, monitored independently by ^1^H and ^25^Mg ^2+^ NMR, suggests that only a single ion binds to the protein in the absence of substrate ^5^. The identified binding site has relatively weak affinity; *K _d_* has been reported in the micromolar ^5^ to low millimolar range ^12^. The second site may be occupied only upon binding of substrate ^8^, possibly due to the presence of high local concentration in the ion cloud of the highly negatively charged nucleic acid molecule. Conformational changes in the active site upon binding the first ion have been suggested as well, with the second site proposed as being responsible for the attenuation of activity at high ion concentrations ^13^. Collectively, these results have been used to propose both a one-metal ^7, 8, 14^ and a two-metal ^10, 15, 16^ catalytic mechanism. Computational work using the quantum mechanics/molecular mechanics (QM/MM) method applied to the *Bacillus halodurans* ^17– 19^ and *Homo sapiens* ^20^ complexes generally supports the two-metal mechanism.

RNase H domains are widely distributed in cellular organisms, but also occur as a component of the reverse transcriptase protein found in retroviruses, in which they are required for viral proliferation ^21^. For this reason, inhibitors of retroviral RNase H domains, particularly that of HIV, have been widely reported ^15, 22^, although none to date have reached clinical use. Most such inhibitors interact with the active site in the metal-bound state ^22^ and therefore must be selective for retroviral RNase H domains to find clinical utility. The HIV RNase H domain (hivRNH) has been reported to bind two metal ions even in the absence of substrate ^23^, although the reason for this difference in behavior between hivRNH and ecRNH is not clear. The *Homo sapiens* RNase H domain (hsRNH) has not been structurally characterized in the absence of substrate and its binding behavior is less well understood. However, it has higher sequence identity and is more structurally similar to ecRNH than hivRNH.

Combined nuclear magnetic resonance (NMR) and molecular dynamics (MD) studies of the behavior of the ecRNH active site residues suggest that the residues of the ecRNH active site are preorganized in the *apo* state for the binding of a single Mg ^2+^ ion ^24^. However, experimental constraints prevent the detailed observation of the protein’s dynamic behavior in the presence of a bound ion at ps-ns timescale. The present work aims to more fully understand the dynamics of ecRNH in the Mg ^2+^-bound state through molecular dynamics simulations of ecRNH in the presence of single Mg ^2+^ ions in various positions in the active site as suggested by crystallographic studies. In addition, the dynamic behavior of the active site in the *apo* state is compared with homologs from other organisms.

## Methods

For each initial protein structure, protonation states for titratable residues were assigned either by experimental measurement (for ecRNH ^3^) or by prediction using the H++ ^26^ pKa predictor. Unless otherwise specified, all simulations were performed at a pH of 5.5 to recapitulate the conditions used in prior NMR experiments on ecRNH ^27, 28^. Crystallographic water molecules were removed from all structures prior to solvation using Schrodinger’s Maestro tool, version 8.5 or 9.1, as distributed in the Desmond software package. Simulations were performed as described ^24, 29^ using Desmond academic release 3 or source release 2.4.2.1 ^30^. Proteins were described with the Amber99SB force field ^31^, solvated with TIP3P water ^32^ in a cubic box with a 10Å buffer region from solute to box boundary, and neutralized with Cl ^−^ ions. Bonds to hydrogen atoms were constrained using the M-SHAKE algorithm ^33^. Simulations containing Mg ^2+^ ions used the Aqvist parameter set ^34^. Electrostatics were calculated with the PME method using a 9Å cutoff. All simulations used a 2.5fs inner timestep on a 1-1-3 RESPA cycle and were performed in the NVT ensemble using a Nosé-Hoover thermostat after equilibration to constant box volume for 5ns in the NPT ensemble. All simulations described in this work were run for 100ns unless otherwise noted. These simulation conditions applied to the *apo* state of RNase H homologs have previously been shown to reproduce NMR data well ^35^.

Order parameters were calculated by the equation ^36^:

$$S^{2}=\frac{1}{2}\left(3\sum_{i=1}^{3}\sum_{j=1}^{3}{\langle \mu_{i}\mu_{j}\rangle }^{2}-1\right)$$

in which *µ _i_* and *µ _j_* represent the x, y, and z components of a unit vector $\vec{\mu }$ in the direction of a given chemical bond. This represents the long-time limit of the angular reorientational correlation function for a given bond vector.

Protein Data Bank (PDB, RRID:nif-0000-00135) structures used for initiating trajectories are listed, along with their resolutions and any system-specific preparation steps, in Table 1.

**Table 1.** Crystal structures used to initiate molecular dynamics simulations of RNases H.

PDB ID	Protein	Source organism	Resolution (Å)	Comments
2RN2 ^37^	ecRNH	*Escherichia coli*	1.48	—
1RDD ^6^	ecRNH	*Escherichia coli*	2.80	*E. coli* WT with single bound Mg ^2+^ ion
1RIL ^38^	ttRNH	*Thermus* *thermophilus*	2.80	—
2E4L ^39^	soRNH	*Shewanella* *oneidensis*	2.00	—
3H08 ^40^	ctRNH	*Chlorobium* *tepidum*	1.60	Missing residues in handle and active-site loop modeled in from 1RIL
2QK9 ^11^	hsRNH	*Homo sapiens*	2.55	Substrate removed and catalytically inactivating D210N mutation reversed in Maestro 8.5
3K2P ^41^	hivRNH	HIV	2.04	Inhibitor and bound metal ions removed in Maestro 9.1; chosen as the HIV structure with lowest RMSD to the unbound state (PDB ID 1HRH) with the active-site loop resolved
3V1O ^42^	xmrvRNH	XMRV	1.88	Full length XMRV RNase H domain with no bound ion
3P1G ^43^	xmrvRNH ∆C	XMRV	1.60	Helix C and handle region deletion mutant of XMRV RNase H domain with single bound Mg ^2+^ ion

Summary of crystal structures used as starting points for molecular dynamics simulations of RNase H homologs.

## Results and discussion

### The crystallographic Mg ^2+^ site is unstable in simulation

**Figure 4.**
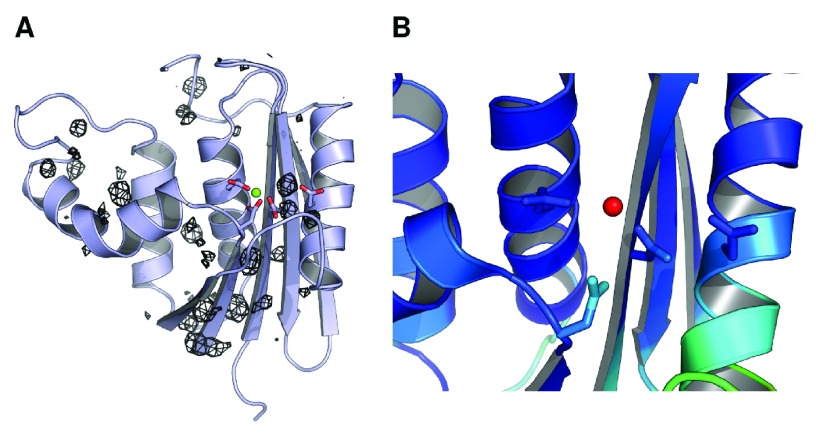
Mg ^2+^ ion dynamics in a simulation initiated from the ecRNH Mg ^2+^-bound crystal structure. ( **A**) Occupancy map from an 89ns simulation initiated from the ecRNH structure solved in the presence of Mg ^2+^ (PDB ID 1RDD), contoured to 0.05% occupancy (corresponding to at least 45ps total residence time). The ion exits the active site and interacts with a variety of regions on the protein surface. ( **B**) The active-site region of the 1RDD structure, colored by atomic B-factor. The B-factor of the ion is substantially larger than the surrounding residues, and is in fact larger than the B-factor of any other atom in the structure save crystallographic waters.

A simulation was initiated from the crystal structure of ecRNH in the Mg ^2+^-bound state (PDB ID 1RDD) ^6^. However, the position of the ion identified in this structure is not stable in simulation and exits the binding site immediately upon initiation of the trajectory. The ion transiently interacts with the protein at a variety of sites on the protein surface over the course of the 89ns trajectory but never returns to its original position in the active site ( Figure 4A).

Historically, simulation of the behavior of multivalent ions using standard molecular mechanics force fields has been a long-standing challenge ^44^. It is therefore possible that the instability of this position in simulation is an artifact of force field errors. However, given that ions in this position are not observed in the substrate-bound structures of RNase H homologs ( Figure 2D), and that the B-factor of the Mg ^2+^ ion in the 1RDD structure is much higher than those of the surrounding residues ( Figure 4B), it is likely that this position does not reflect the most stable conformation of the protein-ion complex in solution.

### Both the crystallographic A and B sites are stable in simulation

Additional simulations were carried out under the same conditions for single Mg ^2+^ ions in each of the two Mn ^2+^ binding sites identified for ecRNH. Because the crystal structure of ecRNH solved in the presence of Mn ^2+^ (PDB ID 1G15) exhibits disorder in both the active-site and handle loops ^7^, the ion positions were instead modeled into the apo ecRNH structure (PDB ID 2RN2) by superposition. For the model of the B-site Mg ^2+^ ion, the rotamer of E48 was also corrected to match the orientation observed in the 1G15 structure. For comparison to an alternative homolog, the Mg ^2+^ ion in the B site was also modeled into the RNase H structure from the thermophilic bacterium *Thermus thermophilus* (ttRNH, PDB ID 1RIL), whose structure was also solved in the absence of divalent ions ^38^.

**Figure 5.**
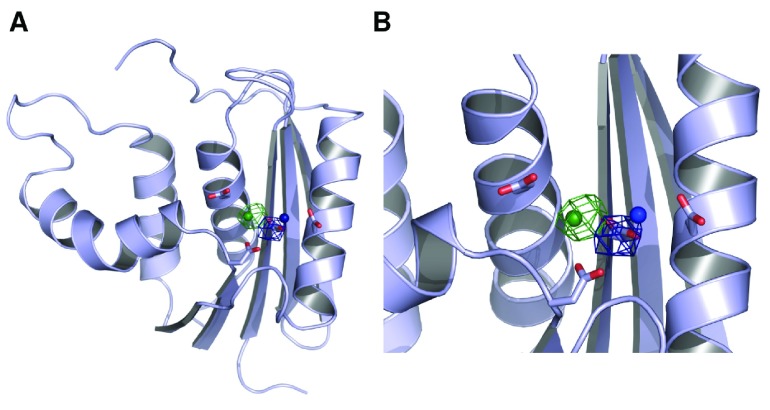
Mg ^2+^ ion dynamics in an ecRNH simulation initiated with single ions in the A and B sites. ( **A**) Occupancy maps contoured at 10% occupancy for the A site (blue) and B site (green). Neither ion leaves the active site over the 100ns trajectory length. ( **B**) Closer view of the active site in which the four active-site residues in apo ecRNH and the positions of the two Mn ^2+^ from which the trajectories were initiated (derived from PDB ID 1G15) are shown for comparison.

Mg ^2+^ ions were found to be stably associated with the ecRNH active site in both simulations, despite the fact that the ions were modeled into a structure that did not originally contain them ( Figure 5). This observation clearly supports the hypothesis that conformational preorganization in the active site promotes ion binding. It is possible that the effectiveness of this modeling procedure was facilitated by a well-documented feature of crystal packing in ecRNH, in which the amino group of a lysine sidechain in a neighboring molecule inserts into the negatively charged active site in a position approximating the B site ^8^. However, a short simulation of ttRNH, whose structure does not contain this contact, with Mg ^2+^ modeled into the B site was also stable, suggesting that crystal contacts in ecRNH are not responsible for the observation of preorganization in its active site.

### The active site is conformationally preorganized for ion binding in the B site

**Figure 6.**
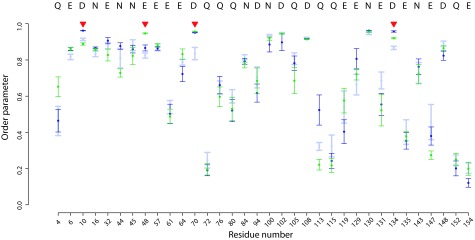
Differences in DENQ sidechain *S* ^2^ values in the presence of Mg ^2+^ ions in ecRNH. Calculated *S* ^2^ values are shown for various simulation conditions with standard errors of the mean: ecRNH apo (light blue), A site (dark blue), B site (green). The four active-site residues are indicated with red triangles. In no case does the difference between any two simulations reach statistical significance.

The presence of Mg ^2+^ located in either the A or the B site did not substantially affect the dynamics of the active-site residues as determined by *S* ^2^. All four residues remain highly rigid in the presence of a Mg ^2+^ ion in either position ( Figure 6). The major difference between the unbound, A site, and B site trajectories’ sidechain dynamics was observed in a short loop between helix D and *β-*sheet 5. Experiments have demonstrated dynamics in this region on the ps-ns timescale, suggesting that the loop is simply incompletely sampled in 100ns simulations rather than significantly perturbed by ion binding. No significant differences in the behavior of these residues upon introduction of ions are observed experimentally ^24^.

Of the four conserved catalytic residues, D134 is known to be somewhat dispensable; catalytic activity is retained, though reduced, by substitutions with N or H, which also increase thermostability ^45^. In conjunction with crystallographic evidence, this suggests that the B site is occupied in the absence of substrate. Because measurements of the sidechain ^13^ *C ^γδ^* resonances by NMR could not clearly distinguish the behavior of D134 (the unique participant in the A site) from E48 (the unique participant in the B site) ^24^, comparisons of the two trajectories provide an additional opportunity to distinguish between these two sites.

Although single metal ions in both sites were stably bound to the protein, the RMSD over the course of each 100ns trajectory was larger for the ion in the A site (1.2Å) compared to the B site (0.6Å), which in turn is similar to the RMSD of a 30ns control simulation of ttRNH with an ion modeled into the B site (0.6Å). Additionally, a small amount of motion in the direction of the B site was observed for the ion in the A site; the initial and final positions differ by 1.7Å ( Figure 5). (By comparison, the A and B sites are about 4Å apart.)

Distinct conformations were also observed for several neighboring residues, reflecting reorganization of local hydrogen bonding networks to accommodate ion binding in each of the two sites. N45 does not differ significantly in sidechain rigidity between the two trajectories, but it does differ in conformation: in the A site trajectory, it is oriented away from the substrate-binding site and participates in a network of interactions that also includes the conserved site T43, while in the B site trajectory N45 is primarily oriented into solvent and occupies the rotamer found in the hsRNH-substrate complex.

**Figure 7.**
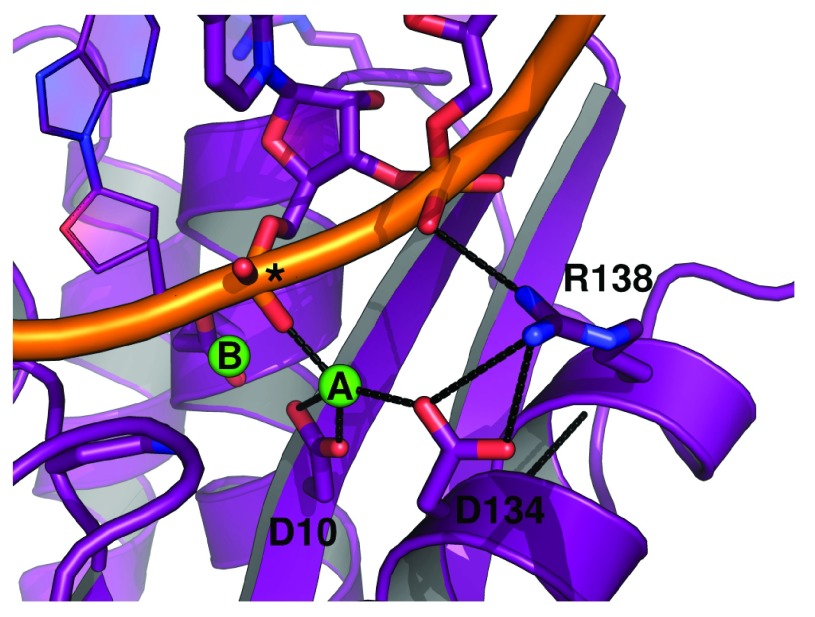
Hydrogen-bonding environment of the A site of hsRNH in complex with substrate. Interactions between D134, R138, metal ion A, and the phosphate backbone of the RNA strand are shown (PDB ID 2QKK). An asterisk indicates the scissile phosphate. The preorganization of the R138-D134 salt bridge in the *apo* state of ecRNH likely minimizes the entropic cost of forming this interaction upon substrate binding.

The hydrogen-bonding network surrounding D134 unsurprisingly differs considerably between the A and B site trajectories. Occupancy of inter-sidechain hydrogen bonds in this region is summarized in Table 2. H124, which interacts with substrate in the hsRNH complex and is known to be associated with product release, forms hydrogen bonds with D134 in the B site trajectory, partially displacing one of the hydrogen bonds formed between D134 and R138 in the *apo* trajectory. By contrast, H124 interacts primarily with E131 in the A site trajectory, while D134 coordinates Mg ^2+^ in a monodentate manner, partially displacing the R138-D134 interaction. This conformation too is at odds with experimental evidence, since E131 experiences minimal chemical shift perturbation upon Mg ^2+^ binding ^24^. Examination of the hsRNH-substrate complex reveals that R138 participates in a hydrogen-bonding network that includes D134 and the phosphate adjacent to the scissile phosphate ( Figure 7); hydrogen-bonding interactions in the apo state may thus minimize entropic costs of binding.

**Table 2.** Hydrogen bond occupancy in the network surrounding D134 in ecRNH.

H-bond	Apo	A Site	B Site
H124-E131	2.2%	47.0%	16.7%
H124-D134	0.8%	1.5%	23.8%
H124-E135	0%	0%	0.3%
R138-E131	0%	11.3%	0%
R138-D134	74.1%	48.4%	65.5%
R138-E135	43.4%	29.8%	50.1%

Comparison of hydrogen-bonding environments in the network surrounding the active-site residue D134 in the apo trajectory of ecRNH compared to trajectories containing an Mg ^2+^ ion in either the A or the B site. Hydrogen bonds were considered formed if the donor-acceptor distance was less than 3.1Å and the donor-hydrogen-acceptor angle was less than 25°.

These results collectively add to prior experimental evidence that the B site is the primary site for metal ion binding in the absence of substrate. Furthermore, the presence of a metal ion in the B site may induce reorganization of the surrounding sidechains into conformations conducive to subsequent substrate binding.

### Rigidity of active-site sidechains is conserved within the RNase H family

Given that all known RNase H homologs have extremely similar active-site structures, it is likely that measurements made on the ecRNH protein can be generalized to other RNase H homologs. *S* ^2^_*MD*_ values were therefore calculated from previous simulations of the four handle-region-containing bacterial RNase H homologs of known structure, as well as for hsRNH in the absence of substrate ^29^.

**Figure 8.**
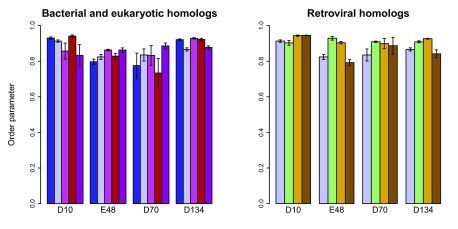
Active-site dynamics in RNase H homologs. Calculated *S* ^2^ values are shown for the four active-site residues in RNase H homologs. Left: soRNH (dark blue), ecRNH (light blue), ctRNH (magenta), ttRNH (red), hsRNH (purple). Right: ecRNH (light blue), XMRV WT (green), XMRV ∆C (yellow), HIV (brown). All simulations were carried out at 300K in the AMBER99SB force field with TIP3P water with structures protonated to reflect a pH of 5.5.

As might be expected from the high level of structural conservation in the active-site region, the five handle-region-containing RNase H homologs compared differ very little in the dynamics of their active site residues ( Figure 8). Notably, the trajectory initiated from the hsRNH structure, which was solved in the presence of substrate and which contained a Na ^+^ ion in a position similar to the B site in ecRNH, differs very little from trajectories initiated from any other RNase H structure lacking these additional components. This observation provides strong support for the interpretation that the rigid active-site residues are conformationally preorganized for metal-ion interactions even in the unbound state.

In order to better understand the relationships between dynamic processes in RNase H domains of retroviral origin compared to those from cellular organisms, additional simulations in the absence of divalent ions were performed on a set of retroviral RNase H homologs. In brief, no significant differences are observed between simulations initiated from the XMRV full-length structure compared to its ∆C mutant (in which helix C and the handle region are removed), between the XMRV ∆C mutant compared to the HIV homolog (which naturally lacks the handle sequence), or between any of the retroviral domains compared to ecRNH ( Figure 8). This result suggests that the preorganization of the active site on the ps-ns timescale is not significantly altered by differences in amino acid sequence, but rather is inherently imposed by the overall protein fold.

## Conclusions

In this work we aimed to use molecular dynamics simulations to understand the dynamic behavior of the RNase H family in complex with catalytically required Mg ^2+^ ions. We observe that the well-studied RNase H homolog from *E. coli* contains a conformationally preorganized active site that is highly rigid on the ps-ns timescale in the presence of a single Mg ^2+^ ion, which is likely located at the B site crystallographically identified by examining the Mn ^2+^ complex. Additionally, we examined the *apo* state dynamics of the active site—previously validated by comparison to NMR data in the case of the *E. coli* homolog ^24^—and found that similar patterns of active-site rigidity are present in all homologs examined, including representatives of bacterial, eukaryotic, and retroviral RNases H. This result suggests that active site dynamics are only subtly modulated by amino acid sequence and are primarily imposed by the characteristic protein fold. Although it has long been recognized that RNases H share similar topologies and active-site conformations with other endonucleases ^46^, the present work extends this observation from static crystal structures to dynamics on the ps-ns timescale.

Simulations of the *Homo sapiens* RNase H homolog and the *Thermus thermophilus* argonaute protein (a distant RNase H homolog with similar active-site architecture) in complex with two Mg ^2+^ ions and substrate analogs also observe high active-site rigidity ^47^; this is consistent with the present data and implies that active-site preorganization is a general property of this larger family of nucleases. Although selective inhibitors of the HIV RNase H domain have been developed based on the hypothesis that the metal ion dependence of the HIV domain’s catalytic mechanism differs from that of the human homolog ^15^, it is likely that the physical origin of this selectivity is not dependent on the active-site conformations sampled on the ps-ns timescale.

## Data availability

ZENODO: Molecular dynamics derived side chain order parameters for Asp, Glu, Asn, and Gln residues in ribonucleases H, and molecular dynamics trajectories for E. coli ribonuclease H. doi:10.5281/zenodo.8431 ^48^
