# Supplementary material for: Evidence from molecular dynamics simulations of conformational preorganization in the ribonuclease H active site
Source: F1000Res. 2014 Mar 7;3:67. [Version 1] doi: 10.12688/f1000research.3605.1 (PMC4032109; doi:10.12688/f1000research.3605.1)
Supplement: Peer review history

# Peer review: Referee response for version 1

**DOI:** 10.5256/f1000research.3859.r4492

**Published:** 2014-05-20

**Authors:** Gaetano Montelione

**Affiliations:** 1 Department of Molecular Biology and Biochemistry, Rutgers University, Piscataway, NJ, USA

This is an exciting paper which beautifully demonstrates the value of MD simulations coupled with experimental data for understanding structure-dynamic-function mechanisms. The research appears to be carefully executed, with proper controls, and the paper is well written. Together with related papers (e.g, Stafford *et al.*, JACS 2014 - ref 24) it would be an excellent topic paper in the area of protein structure and function for an undergraduate or graduate seminar course. 

The results indicate that the apo state of Ec RNase H is conformationally preorganized to favor binding of magnesium ion. MD simulations suggest the primary Mg+2-binding site is actually the observed Mn+2 –binding site B. This is an interesting application of MD to assess the significance of various metal-binding sites previously observed in X-ray crystal structures. 13C nuclear relaxation measurements reveal no statistically significant differences in the fast-time-scale side-chain dynamics of DENQ residues for complexes with Mg+2 modelled into metal binding sites compared with apo Ec RNase H. However, these modelling results suggest some minor structural changes in the active site that may enhance subsequent substrate binding. 

MD simulations from a phylogenetically diverse set of RNase H proteins, including representatives of bacterial, eukaryotic, and retroviral RNAses H, suggest that active sites that are conformationally-preorganized for metal binding are a common feature of the RNase H domain family.

The work is suitable for publication in *F1000Research* but I do suggest minor revisions to address the following points:

1. Fig. 1 shows strong conservation of active site residues among bacterial homogues, and Fig. 8 documents similarity in active site dynamics across a broad phylogenetic distribution, as characterized by MD simulations. The authors conclude that these dynamic properties are only subtly modulated by amino acid sequence and primarily imposed by the characteristic protein fold. As suggested by Reviewer 1 (Rafael Najmanovich), it would be helpful to document the active-site sequence variation across these homologs to support this conclusion.
2. The authors state “ *all simulations were performed at a pH of 5.5 to recapitulate the conditions used in prior NMR experiments on ecRNH*”. What is the pH optimum of enzyme function?  Is there any information about “physiological pH”. Based on the pKa’s of active site ionizable groups, how might the pH selected for this study impact the conclusions.

I have read this submission. I believe that I have an appropriate level of expertise to confirm that it is of an acceptable scientific standard.

# Response

**Published:** 2014-06-20

**Authors:** Kate Stafford

**Affiliations:** Columbia University, USA

Thank you for your thoughtful comments on our paper. In response to your suggestions, we have published a version 2 of the paper which addresses both of your points, specifically:

1. A new Figure 8 has been added to the paper, showing a structure-based sequence alignment for all of the bacterial (soRNH, ecRNH, ctRNH, ttRNH), eukaryotic (hsRNH), and retroviral (XMRV, HIV) RNase H structures used to initiate simulations as part of this work.
2. The pH optimum of the RNase H reaction was previously mentioned only in the Introduction (refs 3 and 4). We have added a comment in the Methods section on the motivation for the choice of pH 5.5 in the NMR experiments and the impact of this on the simulations. In brief, the active site residues are expected to be unprotonated in the presence of Mg2+ at both the experimental pH of 5.5 and the reaction optima of 7.5-9.5.

# Peer review: Referee response for version 1

**DOI:** 10.5256/f1000research.3859.r4005

**Published:** 2014-03-21

**Authors:** Rafael Najmanovich

**Affiliations:** 1 Department of Biochemistry, University of Sherbrooke, Sherbrooke, QC, Canada

The authors set out to use molecular dynamics simulations to the binding of a required magnesium ion on Ribonuclease H1, utilizing a number of distinct structures including those crystallized in different conditions or forms (Apo or different types of Holo forms) and orthologs. The main result found by the authors is that the binding site is very rigid and pre-organized in the presence of a single magnesium ion. The authors show that this observation holds for all orthologs studied. Based on this, the authors suggest that 'the active site dynamics are only subtly modulated by amino acid sequence and are primarily imposed by the characteristic protein fold'. It is indeed interesting that most RNases not only conserve the structure of the binding site but also dynamic properties in the ns-ps range. However, I feel that showing a multiple sequence alignment of the RNase H family highlighting the members used in this study as well as the positions that constitute the substrate and metal binding sites would considerably help understand that indeed this pre-organization comes about despite divergent binding-site sequences. Without seeing such an alignment is hard to accept that the rigidity of the binding-site is not affected by changes in binding-site sequence. If the binding-site sequences turn out to be highly conserved upon analysis of the multiple sequence alignment, it may be necessary to perform mutations in silico in the binding-site to support the conclusion above.

I have read this submission. I believe that I have an appropriate level of expertise to confirm that it is of an acceptable scientific standard.

# Response

**Published:** 2014-06-20

**Authors:** Kate Stafford

**Affiliations:** Columbia University, USA

Thank you for your thoughtful comments on our paper. In response to your suggestion, we have published a version 2 of the paper containing a new Figure 8, which shows a structure-based multiple sequence alignment for all RNases H examined (see reviewer response above). The positions of the active-site residues and the substrate-binding regions have been highlighted on the MSA to facilitate comparison of the sequences within these regions. The point you raise can be subtle - since some sequence similarity is required for maintenance of the overall fold, rather than for specific substrate interactions - so we have edited the text of the Conclusions section for clarity and changed the final sentence of the Abstract to read "...may primarily be imposed...". We have also updated the Conclusions section with a new reference to a recent paper on the evolution of the RNase H superfamily (ref 50).
